# Cortical Visual Impairments and Learning Disabilities

**DOI:** 10.3389/fnhum.2021.713316

**Published:** 2021-10-13

**Authors:** Sylvie Chokron, Klara Kovarski, Gordon N. Dutton

**Affiliations:** ^1^Hôpital Fondation Adolphe de Rothschild, Paris, France; ^2^INCC UMR 8002, CNRS, Université de Paris, Paris, France; ^3^Department of Vision Science, Glasgow Caledonian University, Glasgow, United Kingdom

**Keywords:** learning disability, assessment, differential diagnosis, CVI, occipital

## Abstract

Medical advances in neonatology have improved the survival rate of premature infants, as well as children who are born under difficult neurological conditions. As a result, the prevalence of cerebral dysfunctions, whether minimal or more severe, is increasing in all industrialized countries and in some developing nations. Whereas in the past, ophthalmological diseases were considered principally responsible for severe visual impairment, today, all recent epidemiological studies show that the primary cause of blindness and severe visual impairment in children in industrialized countries is now neurological, with lesions acquired around the time of birth currently comprising the commonest contributor. The resulting cortical or cerebral visual impairments (CVIs) have long been ignored, or have been confused either with other ophthalmological disorders causing low vision, or with a range of learning disabilities. We present here the deleterious consequences that CVI can have upon learning and social interaction, and how these can be given behavioral labels without the underlying visual causes being considered. We discuss the need to train and inform clinicians in the identification and diagnosis of CVI, and how to distinguish the diagnosis of CVI from amongst other visual disorders, including the specific learning disorders. This is important because the range of approaches needed to enhance the development of children with CVI is specific to each child’s unique visual needs, making incorrect labeling or diagnosis potentially detrimental to affected children because these needs are not met.

## Introduction

Vision is fundamental to learning. Sight guides our limb and body movements. It also provides access to a vast range of information, and facilitates social interaction. Children are not only continually learning these skills, they also learn through these developing abilities.

Cortical or cerebral visual impairments (CVIs) include a wide range of visual dysfunctions that can impair learning and social interaction. The present review describes CVI and provides examples helpful to a range of professionals dealing with children with learning disabilities including pediatricians, child psychiatrists, and child neuropsychologists. Learning disabilities refer to brain conditions impairing the capacity to learn in several areas, for which the cause has yet to be identified. A learning disorder or difficulty is commonly “diagnosed” in children presenting significant delay in their development of several functions. Formal diagnoses include intellectual disabilities, specific learning disorders (affecting reading, writing, and mathematics) but also motor learning disorders (American Psychiatric Association, 2013). Crucially, these are often associated with neurodevelopmental conditions, making it urgent to identify and diagnose the underlying causes, and risk factors. This review offers an overview to consider how the diagnosis of CVI can potentially explain, at least in part, a wide range of learning difficulties, which can be overcome by appropriate management and educational strategies made accessible to the affected child.

## The Differences Between Typical Vision and Cortical or Cerebral Visual Impairment

Picture a first-year schoolboy coming home from school, running into the kitchen, climbing onto a chair, and reaching into a tin for a biscuit. What part does vision play? He mentally envisions within his frontal territory what he wants to do and how (Buckner et al., 2008). He rapidly uses visual memory to navigate to the kitchen (Sanguinetti and Peterson, 2016). His eyes focus automatically by means of the lenses accommodating. His retinae turn the incoming imagery into unique patterns of electrical activity, with each glance capturing new imagery to integrate into a seamless pictorial flow (Churan et al., 2018). The optic nerves continuously transfer these signals via his lateral geniculate bodies to his occipital lobes, where analysis of the structure of the scene, in terms of extent, clarity (acuity), brightness, contrast and color takes place within about a tenth of a second (Lesniak et al., 2017), while the adjacent middle temporal lobes capture the flow of movement of the scene (Zihl and Dutton, 2015).

This processed information is immediately transferred to the temporal lobes via a bundle of nerve fibers on each side, called the inferior longitudinal fasciculi known functionally as the ventral stream (Bauer et al., 2015) dealing with local, detailed, visual processing, wherein a match with the coded library of past imagery brings about recognition of the tin. At the same time, the occipital lobes pass the processed image data to the posterior parietal lobes, via the superior longitudinal fasciculi (Bauer et al., 2015), functionally known as the dorsal stream dealing with global visual processing. This process is supported by the middle temporal lobes (which supplement the kinetic flow of the moving scene), and the deeper brain structures, the posterior thalamus and superior colliculi (Ptito et al., 2008), which together bring about non-conscious 3D mapped mental emulation of the scene, facilitating visual search and visual guidance of movement. There is evidence that the mapping of sound localization takes place in the same brain region (Thaler et al., 2016).

This mental visual construct enables the chair to be located and dragged to the right place, climbed onto, and the biscuit retrieved. The boy also recruits his cerebellum to modulate the timing of his actions, as well as his balance (or labyrinthine system) to climb onto the chair. In the inner ear there are balance receptors. Minute lumps of calcium linked to nerve endings to detect gravitational forces, act as plumblines, integrating with his vision (Jayakaran et al., 2018) through his semi-awareness of the horizontal edge of the kitchen wall cabinet, automatically ensuring his stability. In essence, through this highly efficient real-time process, the boy’s mind processes a continuously flowing emulation of the surrounding moving scene, mapped to his body, enabling him to recognize, and integrate and interact with his surroundings.

Disturbance of any element of these complex mental visual processes can occur in a range of patterns of CVI, unique to each child. These need to be identified, characterized and profiled to provide matched habilitational approaches designed to cater for each element of the resulting visual and associated disabilities.

### Definitions, Epidemiology, Etiology of Cortical or Cerebral Visual Impairment

#### Definitions

Cortical or cerebral visual impairment can be defined as “a verifiable visual dysfunction, which cannot be attributed to disorders of the anterior visual pathways or any potentially co-occurring ocular impairment” (Sakki et al., 2018). This broad consensus definition embraces the wide range of damage or dysfunction of the neural pathways, centers and networks involved in visual information processing. Children with CVI have been sub-classified into those who show selective visual perception and visuo-motor deficits, those with more severe and broader visual perception and visuo-motor deficits, and those with profound visual impairment (Lueck and Dutton, 2015b; Sakki et al., 2021).

These disorders compromise any of the following aspects of visual function in a range of combinations: central vision, peripheral vision (in all or part of the visual field), movement perception, gaze control, visual guidance of movement, visual attention, attentional orientation in space, visual analysis and recognition, visual memory and spatial cognition. Affected young children “know” their vision to be “normal,” yet the educational, developmental, and emotional personal and social impact of living with unreliable perception is commonly profound.

#### Epidemiology

In epidemiological terms, CVI has become the leading cause of major visual impairment in industrialized countries (Kong et al., 2012). This change can be linked to improvement in the survival rates of children born prematurely and/or those with neurological damage, as well as better prevention of visual deficits of ocular origin. CVI in children is common, potentially affecting at least 3.4% of children but many affected children go unidentified (Cavezian et al., 2010a; Williams et al., 2021). The proportion of children with learning difficulties attending special schools who have CVI is high (Black et al., 2019) and may be greater than 50% (Williams et al., 2021).

#### Etiology

As with other neurodevelopmental conditions (e.g., autism spectrum conditions, learning disabilities, ADHD), children born with complex neurological conditions are at risk of developing CVI. Indeed, complications of premature birth and perinatal cerebral anoxia (or hypoxia), are the most frequent causes of CVI (Fazzi et al., 2009). Other common etiologies include head injury, stroke, brain infection and genetic neurodevelopmental disorders (Lueck and Dutton, 2015a). CVI results from lesions affecting the posterior visual pathways, the optic chiasm, lateral geniculate bodies, optic radiations, primary visual cortices, the middle temporal lobes (serving movement perception) and the visual association areas. The visual functions of these structures can be affected to varying degree, either in isolation or in a variety of combinations. When the thalamus is involved, the lack of vision tends to be profound (Ricci et al., 2006). Moreover, the resulting visual impairment may be exacerbated by disorders of eye movement control (Fazzi et al., 2009; Boot et al., 2010; Ortibus et al., 2011; Lueck and Dutton, 2015b). CVI is therefore an umbrella term referring to visual deficits not specifically related to ocular, optic nerve or chiasmatic damage, but to pathology behind the chiasm, in particular affecting the visual brain areas involved in integration, identification, analysis and interpretation of static and moving visual information, as well as in visual control of directed movement in the environment.

Typical clinical features of CVI may be manifest in a child, despite undetected brain signatures. Even adults with visual field loss following stroke, can show normal MRI brain imaging in 30% of cases (Zhang et al., 2006a,b; Kelly et al., 2021), as can around 12% of children with Cerebral Palsy (CP) (Robinson et al., 2009; Towsley et al., 2011). It is therefore important to acknowledge that a report of a “normal” brain MRI in a child with neurovisual impairment does not exclude the diagnosis of CVI.

The term “minimal (or mild) brain injury” is sometimes used to refer to brain dysfunction in these children. Yet the consequences of the resulting visual difficulties and their impact on learning are far from “minimal” having far-reaching implications for the child’s learning, motor, cognitive and social development (Chokron and Dutton, 2016) with the effects on quality of life being akin to those of lack of primary visual functions (Mitry et al., 2016).

#### Optical, Ophthalmological and Neurological Disorders Associated With Cortical or Cerebral Visual Impairment

Cortical or cerebral visual impairment can occur in isolation or in association with eye or optic nerve damage (Jacobson and Dutton, 2000; Fazzi et al., 2007). Moreover, around 50% of children with CVI have refractive error or impaired focusing (hypoaccommodation), necessitating spectacle correction (Pehere et al., 2018), so all such children need to have their range of accommodation checked (by dynamic retinoscopy) and must be refracted and have their post-refraction vision checked with their salient spectacle correction for both near and distance to plan their habilitation.

Lesions affecting the optic radiations lead to detectable ganglion cell absence in predictable retinal areas owing to a process known as retrograde transynaptic degeneration (Lennartsson et al., 2014) with lack of the optic nerve fibers causing optic atrophy or optic disk cupping which can be misdiagnosed as glaucoma (Jacobson et al., 2020), when brain injury occurs in later pregnancy (Jacobson and Dutton, 2000) or optic nerve hypoplasia, as a sequel to earlier injury (Zeki et al., 1992).

Children with CVI are frequently observed to have oculomotor disorders, difficulties in visual fixation or visual pursuit, hypometric saccades, or a disorder of gaze strategy (Stiers et al., 2002; Fazzi et al., 2004), as well as nystagmus due to periventricular leukomalacia (Jacobson et al., 1998; Tinelli et al., 2020). These conditions are associated with reduced visual performance.

Some children with CVI show academic success similar to their typical peers, while others show significant learning disabilities. Early brain damage is commonly diffuse, so tends to affect multiple brain functions, leading to associated neurological disorders including epilepsy, intellectual disability and CP, which can compound the deleterious effects of CVI on development (Lowery et al., 2006; Duke et al., 2020). Several studies have been conducted in children with CP to identify and characterize their associated CVI (Stiers et al., 2002; Fazzi et al., 2004; Pehere et al., 2018). These investigations have shown that children with CP commonly have difficulties in visuo-perceptual, visuo-spatial and visuo-constructive activities, regardless of their level of visual acuity (West et al., 2021). The severity and patterns of the deficits closely correlate with the extent and distribution of reduction of white matter as well as impairment of the dorsal stream pathway, interfering with attentional, spatial and motor aspects of visual cognition as well as with global visual processing (Fazzi et al., 2004; Duke et al., 2020). MRI tractography has shown that when the inferior longitudinal fasciculi in periventricular temporal lobe white matter are affected, the ventral stream dysfunctions alter detailed visual processing and in this way, visual recognition (Ortibus et al., 2012), and when the superior longitudinal fasciculi are affected, the resulting dorsal stream dysfunctions impair visual mapping of the visual scene, leading to simultanagnostic vision limiting visual search, with lack of accuracy of visual guidance of movement (optic ataxia) (Bauer et al., 2014) (see below for a detailed description).

#### Patterns of Cortical or Cerebral Visual Impairment

Many patterns of visual disorder can be seen, with each affected child having their own unique form of vision (Philip and Dutton, 2014). Depending on the topography and extent of the pathology, the deficit may impair any aspect of visual function affecting central vision, peripheral vision (in all or part of the visual field), movement perception, gaze control, visual guidance of movement, visual attention, attentional orientation in space, visual analysis and recognition, visual memory and spatial cognition, as well as central vision, the visual fields, visual analysis, visual exploration, visual attention, or visual memory (Kelly et al., 2021), in any combination, or degree. Recognition or visual memory of an object, a face or a place, the act of processing a set of stimuli or a complex scene, or difficulties directing movement or gesture under the control of vision, can be impaired in a variety of combinations. When the optic tracts, lateral geniculate bodies, optic radiations, or primary visual cortices are affected by a lesion, the resulting CVI manifests as lack of vision for all or a portion of the visual field.

Considering central vision, corrected visual acuities of children with CVI can be normal, subnormal or profoundly impaired. Contrast sensitivity perception is often significantly impaired (Good et al., 2012), while anomalous light brightness appreciation is a likely cause of photophobia, but the effects of CVI on perception of color have yet to be systematically studied.

Observed visual field deficits range from cortical blindness (i.e., lack of all visual sensation despite the integrity of the eye) to scotoma (i.e., lack of visual sensation for a small portion of the visual field). Intermediate disorders include tunnel/tubular vision (i.e., concentric reduction of the visual field), or its opposite, retention of peripheral vision (i.e., loss of the central visual field, while the peripheral visual field is preserved), homonymous lateral hemianopia (i.e., loss of the contralesional visual field), or quadrantanopia (i.e., loss of a visual quadrant). Lower visual field impairment due to periventricular leukomalacia (often associated with premature birth or CP) can be peripheral or complete, or manifest as degraded clarity in the lower visual fields (Jacobson et al., 2006; Tinelli et al., 2020), and it can be combined with dorsal stream dysfunction, leading to a major deficit in global visual processing. These different disorders may exist as such or be observed successively in the same patient who may show a degree of recovery over time (Guzzetta et al., 2001b; Watson et al., 2007; Werth, 2008).

The lower visual field impairment (which if peripheral, may not be detected by classical central visual field testing) is characterized by adaptive strategies of walking with the head down, tripping over obstacles, reluctance to jump off a bench, holding onto clothing of an accompanying adult (while pulling down) to provide tactile guidance for the height of the ground ahead when walking over uneven ground, going down stairs by running the heel down the riser, and probing the ground ahead with the foot to check whether a floor boundary is a step or not (Lueck and Dutton, 2015a). Reaching in the upper intact visual field is often more accurate than in the lower visual field, when it is impaired. The accompanying dorsal stream dysfunction often leads to distress in crowded and noisy locations, inability to find an object in clutter, or a friend in a group, or to read, unless peripheral text is masked. While looking away from a face into an uncluttered area when listening to someone speaking, to facilitate auditory attention is also common (Zihl and Dutton, 2015; Dutton et al., 2017).

Expansion of the lateral ventricles into temporal lobe white matter in children with hydrocephalus can implicate a pattern of evident ventral stream dysfunctions, such as impaired processing of visual details, impaired visual recognition of faces and facial expressions, as well as difficulties with navigation, and object and word recognition. Shunted hydrocephalus, leading to CVI in 50% of cases, is a cause of this pattern of vision (Houliston et al., 1999; Andersson et al., 2006).

Children with quadriplegic CP can be similarly but more profoundly affected. Complete lower visual field impairment from severe posterior parietal injury, combined with hemianopia from asymmetric cerebral hemisphere injury, may culminate in a single intact upper visual field quadrant of intact vision only. This needs to be sought out and optimally utilized for communication and learning. Associated severe dorsal stream pathology due to bilateral posterior parietal pathology can result in apparent blindness owing to additional probable Balint syndrome (see section “Cortical or Cerebral Visual Impairment, Visuo-Motor Coordination and Gesture Production”). Yet, elimination of all visual and auditory “clutter” by enclosing such children in a monochrome “tent” for a succession of half hour periods can lead to visual behaviors gradually becoming manifest for the first time, even in older children, which can later be sustained even outside the tent (Little and Dutton, 2015).

### Semiology of Cortical or Cerebral Visual Impairment in Children

#### Cortical Blindness, Visual Field Defects and Blindsight

**FIGURE 1:**
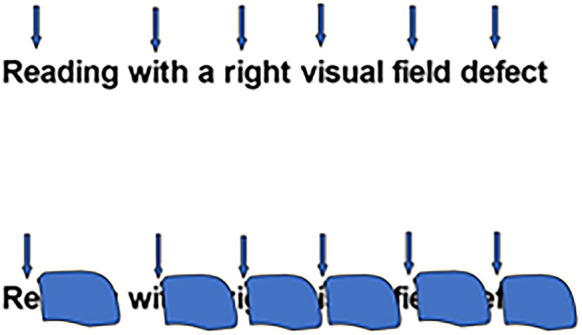
Reading with a right sided visual field defect. **Above:** Upper arrows denote the position of the fovea with respect to the word when reading. **Below:** position of the right scotoma overlying the word when reading.

Lesions in the optic tracts, lateral geniculate nucleus, optic radiations or primary visual cortex result in loss of vision in all or part of the visual field (depending on the location and severity of the lesion). The observed visual-field defects range from cortical blindness (i.e., loss of all visual sensation despite the integrity of the eye) to scotoma (i.e., loss of visual sensation in part of the visual field). Moderate impairments include tunnel vision (i.e., a concentric reduction in the visual field; see Figure 1) or conversely, peripheral vision (i.e., loss of central vision only). Some children are born with these impairments, whereas others acquire them at a later stage (Lueck and Dutton, 2015b).

Visual-field defects among children are defined by a loss of visual sensation in all or part of the visual field. Unfortunately, there is little public awareness of visual-field defects and they are not usually tested for clinically, whereas, curiously, ocular damage is diagnosed and treated early on. Thus, there is a profound lack of knowledge on cerebral visual deficits in children. Unfortunately, in pediatric patients the sequelae of neonatal cortical blindness are far too frequently diagnosed late [often around the age of 10, by which time the child has already completed several years of school, and sometimes after testing for a pervasive developmental disorder (PDD)] (Lueck et al., 2019).

In terms of signs and symptoms, the initial phase of cortical blindness typically involves loss of all conscious visual sensation as well as loss of the blink reflex to light or to visual threat. Children with such deficits behave as if they are blind, avoiding obstacles and people; they cannot even make basic distinctions between light and dark or between motion and stillness. However, this situation only lasts a few weeks: the child eventually recovers basic visual function, although this can be limited to a diminished visual field, where they only detect high-contrast or moving visual stimuli. Due to delayed diagnosis, children who suffer cortical blindness are often examined only several years after onset of their lesion. They exhibit a less classical set of signs and symptoms than do adults in the acute phase. However, despite the time that has passed since lesion onset, these children typically show a lack of interest in visual stimuli and have marked difficulties in fixing their gaze on such stimuli.

For children who have grown up with cortical blindness that they acquired during the neonatal period but who are evaluated only at an older age, the term *cortical blindness* is generally inappropriate (Watson et al., 2007; Werth, 2008). In these children, the sequelae of cortical blindness manifests as partial bilateral visual field defects such as tunnel vision (perception within a 10–20° concentric area in the central visual field) or peripheral (absence of perception in the central visual field) often accompanied by other visual cognition disorders such as simultanagnosia, visuo-motor ataxia, disorder of orientation of attention in space (Kelly et al., 2021), as well as disorders of visual recognition of objects and/or faces. Children with profound CP suffering from cortical blindness who are first seen with visual difficulties years after the onset of their deficit (owing to lack of screening at birth) present a less clear picture than that of the adult in the acute phase of bilateral occipital infarction. Even long after presentation, a reduced interest in visual stimuli can be observed, with great difficulty in mobilizing gaze toward visual stimulation. In spite of this, light and sound stimuli presented in the dark can trigger eye movements or visual fixation, which is often not evident in ambient light. It is important to note that, similar to adult patients, children with CVIs can also exhibit a dissociation between their abolished conscious perception and a type of non-conscious perception, known as *blindsight*, which enables them to avoid obstacles and process visual information in their blind visual field without being aware of doing so (see Weiskrantz, 2004 for an extensive discussion on this phenomenon). According to Tinelli et al. (2013), conversely to children with acquired lesions and CVI, residual unconscious processing of position, orientation and motion of visual stimuli displayed in the scotoma of children with congenital lesions and CVI (Tinelli et al., 2013). We have occasionally seen children with sustained bilateral occipital lobe infarction, who sometimes manifest appropriate responses to smiles suggestive of affective blindsight (Celeghin et al., 2015). Other children can show remarkably good mobility despite their very low vision, probably due to intact middle temporal lobe function causing the Riddoch phenomenon as described in adults (Arcaro et al., 2019), allowing them to distinguish moving stimuli in the blind visual field (Boyle et al., 2005; De Agostini et al., 2005; Tinelli et al., 2013). Unfortunately, these visual field impairments can be completely missed, partly because the child is unaware of his deficit, and partly because the disorder is not visible to the clinician, and can only be identified by seeking it out (Pawletko et al., 2014).

#### Visual Cognition Deficits

Ventral and dorsal stream disorders give rise to more complex perceptual conditions, as described below.

##### Ventral stream dysfunction

Ventral stream dysfunction tends to result from temporal lobe pathology, leading to impaired visual analysis, visual recognition and route finding, while dysfunction of the middle temporal lobes can lead to impaired perception of movement (or dyskinetopsia), degrees of which are common in children born prematurely, especially if they have periventricular leukomalacia (Guzzetta et al., 2009).

Visual and spatial imagery disorders are commonly seen in clinical practice (for review see Tanet et al., 2010). These can be highlighted through tasks such as producing and copying geometric figures, arranging cubes, solving puzzles and mental imagery tasks (i.e., “visualizing a representation” needed to answer a question about the characteristics of the object). These approaches have yet to be systematically described in the literature.

Visual recognition disorders (known as visual agnosia in adults) are the result of damage to the occipito-temporal region and are not related to impaired verbal skills. Because learning visual information is almost impossible, the child has difficulty interpreting what is seen, but retains recognition through another sensory modality (e.g., touch). The most frequent recognition difficulties concern images and objects (Tanet et al., 2010; Pawletko et al., 2014). However, these difficulties may also concern faces and sometimes even reading and spelling (for review see Fazzi et al., 2009).

##### Dorsal stream dysfunction

Dorsal stream dysfunction results from posterior parietal pathology limiting parallel processing of multimodal mental mapping of the surroundings owing to attenuation of the superior longitudinal fasciculi (Bauer et al., 2015). This impairs visual exploration and limits attention through simultanagnostic vision (difficulty recognizing objects when presented simultaneously but with preserved ability to recognize them separately). It also impairs the dorsal stream non-conscious mental mapping of motoric space, leading to inaccurate visual guidance of movement (optic ataxia, which is characterized by the difficulty directing voluntary acts under the control of vision) leading to impaired visuo-motor coordination (Atkinson and Braddick, 2020). When severe, this manifests as Balint syndrome, which together with unilateral spatial neglect (also known as neglect or hemifield inattention) have been described in children (Gillen and Dutton, 2003; De Agostini et al., 2005; Drummond and Dutton, 2007; Philip et al., 2016).

Balint syndrome comprises three main clinical signs (Rizzo, 1993). First, what Balint called “psychic paralysis (or apraxia) of gaze” which refers to an inability to voluntarily redirect gaze to a nominated target. Second, simultanagnosia which corresponds to a restricted field of visual attention and finally, optic ataxia, that is a major deficit of visuo-motor coordination. Balint Syndrome is observed following bilateral parietal brain injury, but each of the features may be evident in less extensive lesions such as those resulting from subtle posterior superior periventricular leukomalacia, often associated with premature birth (Saidkasimova et al., 2007). This form of CVI is the commonest variant we have observed (Dutton et al., 2004).

Unilateral spatial neglect, most often evident when it affects the patient’s left-side as it tends to be more severe on this side, is characterized by difficulties in reacting to, or acting upon stimuli presented in the hemispace contralateral to the brain lesion. This deficit, in which the patient behaves as if half of space on one side does not exist, can be observed in visual and manual activities (e.g., searching and reaching), but also at the locomotor level (e.g., showing a tendency to turn only toward the non-neglected side) (De Agostini et al., 2005). Clinically, head and eye rotation to the left does not compensate for the resulting left sided inattention, but body rotation does, indicating that the posterior parietal map of the surrounding environment is egocentric (Chokron et al., 2007).

Blurred vision or lack of visual field due to CVI may also impair visuo-motor coordination because the low vision is insufficient to allow movement to be accurately visually guided, giving a false impression of clumsiness. Typically, affected children’s performance of gesture and action are more accurate when tactile and kinesthetic input is used in favor of vision (for review and discussion see Stiers et al., 2002).

## Behavioral Expressions of Cortical or Cerebral Visual Impairment in Children

Not only are the CVIs not clearly visible because the ocular system appears normal, but also, children growing up with CVIs due to ante- or neonatal injury have no way of knowing their vision is not “normal.” This is not a genuine anosognosia, or a lack of awareness, but an actual inability for the child to recognize her vision is disordered. This means that CVI is not consciously symptomatic. As a result, CVI often goes unidentified. Rather the deleterious consequences on behavior, learning or interactions alert parents, teachers and clinicians that something is amiss. This undoubtedly explains why this disorder is under-diagnosed and why it can be confused with other conditions such as autism, coordination acquisition disorders or learning disabilities (Chokron and Dutton, 2016; Chokron et al., 2020). In turn, the existence of undiagnosed CVI also explains the inflation of other default diagnoses in these children, such as behavioral or learning disorders (Lueck and Dutton, 2015b; Zihl and Dutton, 2015). Table 1 summarizes a number of situations in which CVI can only be indirectly expressed (Chokron and Dutton, 2016).

**TABLE 1:** Main behavioral expressions of CVI in children.

**Behaviors**	**Possible visual explanations**
Failing to look the speaker in the eyes (lack of eye contact) but looking to the side. *Diagnostic confusion with a severe interaction disorder (e.g., autism).*	Simultanagnostic vision Visual field deficits (e.g., tunnel vision) Cortical blindness
Fragmented vision of the world, loss of the target, panicky fear of being lost or not being able to find a parent. Fear of approaching people and crowded places. *Diagnostic confusion with a severe interaction disorder (e.g., autism).*	Visual field deficits (e.g., tunnel vision) Simultanagnosia Balint Syndrome Visual extinction
Not seeing an object in front or in the near visual field. Often moving the head and eyes to compensate for the blind visual field. May hold head tilted down Neglecting a part of the visual information (e.g., eating from only part of the plate, writing on only part of the page). *Diagnostic confusion with an attention deficit disorder, clumsiness or a practical problem.*	Visual field deficits Spatial neglect
Difficulties to visually control a gesture, graphic clumsiness, difficulties in copying, clumsiness for gestures requiring fine visual-motor coordination (e.g., Picking up a very small object on a textured mat yet can run into an approaching obstacle when distracted by talking). *Diagnostic confusion with a practical disorder or a coordination acquisition disorder.*	Visual field deficits Deficit of ocular movements control Visual exploration deficit Visuo-manual coordination deficit (optic ataxia) Balint Syndrome and dorsal stream dysfunction
Not recognizing large letters but perceiving very small letters. *Incomprehension by family and school. (It can be incorrectly diagnosed as functional visual impairment.)*	Global visual perception deficits. Simultanagnosia Visual field deficits (e.g., tunnel vision)
Misuse of objects, difficulties with autonomy (dressing, eating, looking for a specific object) *Diagnostic confusion with intellectual disability or autism.*	Visual recognition deficit Simultanagnosia Optic ataxia
Difficulty analyzing a set of visual stimuli on a page. When reading, inability to process all letters, syllables or words. *Diagnostic confusion with dyslexia.*	Refractive error Dysfunctional accommodation Low visual acuities Eye movement disorders Simultanagnosia–Balint Syndrome Visual extinction
Difficulty constructing and evoking a mental image. *Recognition difficulties, lack of mental imagery, difficulties drawing from memory.*	Visual memory defect
Impossibility to follow rapid movements (e.g., fleeting facial expressions, rapid demonstrations, movements made by babies or animals). Difficulty in appreciating distances and fear of being struck by an object or person. *Isolation in the schoolyard and diagnostic confusion with an interaction disorder (e.g., autism).*	Dorsal stream dysfunction Dyskinetopsia Visual motion processing deficit Spatial orientation deficit
Getting lost, not recognizing known places. *Search for rituals to avoid disorientation and withdrawal (e.g., autism)*	Spatial orientation and spatial representation deficits Place recognition deficit
Getting lost when aligning numbers in an operation, lack of a numerical mental line, difficulty in visualizing order of magnitude and time. *Diagnostic confusion with dyscalculia*	Spatial organization and coordination disorder Spatial neglect Dorsal stream dysfunction Balint Syndrome

Cortical or cerebral visual impairment can take various forms and is expressed in daily life in multiple ways, hindering development, social adaptation, learning and social interaction (Chokron and Démonet, 2010). Most often, clinicians and parents focus on these highly evident manifestations, which are the consequences of CVI, but not on the CVIs themselves. For example, a child can be mistaken for suffering from dyslexia if there is consequent difficulty reading, or as having developmental coordination disorder if fine visual-motor coordination interferes with tasks, without recognition that CVI is the origin of the reading or motor difficulties.

### Cortical or Cerebral Visual Impairment and Learning Disabilities

No-one can learn from information they cannot perceive. Children with impaired vision are unaware of what they do not see. Visual deficits due to CVIs likely impair learning in many children worldwide, simply because their visual needs are not being catered for. We all adapt to the circumstances we find ourselves in, and children with CVI, (whether or not their CVI has been identified) are no exception. If we cannot see something, we cannot respond to it. If an event is stressful, frightening or objectionable, we react emotionally to it, but when we have the capacity to overcome circumstances, we adapt our behavior accordingly. Children with CVI are no different and manifest the self-same patterns of behavior, as the natural consequences of the way they perceive their worlds, which of course is their normal. Such conditions may be seen as “behavioral disorders,” when in fact they are signatures for the now well-known underlying diagnosis of CVI. Indeed, vision plays an essential role in the development of sensory-motor and cognitive abilities (Atkinson and Braddick, 2007; Chokron and Dutton, 2016). It provides the facility to coordinate all the sensory-motor systems (Fraiberg, 1977). Visual experiences are the first involved in the development of mental representations (Warren, 1994; Fazzi et al., 2010), which will later be crucial for the development of concepts and abstraction. Vision also allows the child to learn through imitation, a process essential to human development.

Although studies on the impact of CVI on the development of young children are rare, there is a large literature on the effect of ophthalmologic visual impairment on development. Studies conducted in blind children, for example, report a marked delay in all areas of motor development compared to sighted children (Fraiberg, 1977; Sonksen, 1993). In a very similar way, children with multiple neurological disorders, such as those with CP, often manifest delay in postural development (Fraiberg, 1977), as well as difficulties in acquiring object permanence, which can be interpreted as a marker of the level of cognitive development (Fazzi et al., 2011). In children with CVI, the problem is even more complex because their perceptual disorders are most often defined and “diagnosed” without questioning their origin, nature or severity.

While there is no question of attributing all learning or behavioral disorders to visual function disorders, it is obvious that, conversely, given the role of vision in development, children with CVI are at significant risk of developmental disorders affecting the entire cognitive and social sphere, as described below. It is therefore crucial to establish the differential diagnosis between CVI and learning disorders, even if this has yet to be rendered systematic policy. Indeed, apart from neuropsychological disorders directly related to neurological injury, CVIs are likely to hinder the development of different skills and learning, as well as interfering with the way the child interacts with the world. It is common to observe that a child suffering from CVI involving visual field, visual attention, or visual analysis, commonly manifests learning, behavioral and/or social interaction disorders as a consequence (Jacobson and Dutton, 2000; Fazzi et al., 2009; Pawletko et al., 2014). Impairments in these functions may manifest as difficulties in reading, in coordination and in social interaction.

#### Cortical or Cerebral Visual Impairment and Reading

Word identification during reading is possible thanks to the great clarity of our central vision, served by the foveal zones of the retinae. However, reading also involves the use of clues in the para-foveal zones, i.e., in the area adjacent to the central visual field. A visual field disorder affecting all or part of the para-foveal field will therefore inevitably alter the quality of reading (see Figure 1).

In fact, homonymous hemianopia is accompanied by a considerable slowdown and hesitancy in reading fluency as well as anomalies in the amplitude and latency of ocular saccades toward the two visual fields (contra and ipsilateral) (Fayel et al., 2014). On the other hand, several authors have shown the role of attention in reading skills, and even more so in learning, for which it has been shown that visual attentional skills are among the prime predictive factors (Plaza and Cohen, 2007). Thus, a massive attention deficit such as unilateral spatial neglect may be accompanied by neglect dyslexia, where reading errors will involve the neglected (usually left) part of the text and/or words (Laurent-Vannier et al., 2003; Lee et al., 2009; Chokron and Cavezian, 2011). Although reading disorders are not systematically associated with signs of unilateral spatial neglect, children with left-sided neglect may omit or substitute the left part of a text, the beginning of sentences, and have great difficulty returning to the line (Ellis et al., 1987).

Another attentional disorder that may impair reading skills is simultanagnosia. This deficit, in which the patient sees only singular elements, can limit the ability to group the letters seen, and consequently prevent the correct grouping of letters to make up the word.

Finally, CVI can also alter reading and learning due to the presence of a disorder in the recognition of spelling material. Apart from letter-by-letter reading that seems to be acquired, this recognition disorder seems to make it impossible to build up the lexical stock (by inability to recognize syllables and/or words) and is the cause of difficulty in learning to read. It is interesting to note that the case reported by O’Hare et al. (1998) shows that such a form of alexia may exist in children and that it may be the direct consequence of an occipital lesion.

#### Cortical or Cerebral Visual Impairment, Visuo-Motor Coordination and Gesture Production

Processing of visual information plays a key role in the design, control and execution of movement, especially manual skills (Costini et al., 2014a). Indeed, vision serves as the first support for learning postural control, and it is only at the next stage of learning that the child comes to use tactile and vestibular information (Guzzetta et al., 2001a). Consequently, a CVI may alter an individual’s psychomotor skills in the form of optic ataxia (Hay et al., 2020) and can easily be confused with a practical disorder or dyspraxia (Chokron and Dutton, 2016). At the same time, just as unilateral spatial neglect has been associated with motor difficulties such as akinesia or hypokinesia in adults, current evidence suggests that CVI in children, and in particular unilateral spatial neglect, are most often associated with motor neglect as well as with praxis disorders (Gaudry et al., 2010; Chokron and Dutton, 2016). In addition, optic ataxia is defined as a specific difficulty in directing a ballistic gesture under the control of vision. This disorder therefore specifically affects visuo-manual and visuo-motor coordination and is characterized by difficulties in directing voluntary and coordinated acts under the control of vision (Gillen and Dutton, 2003), particularly for pointing and grasping activities.

Finally, it should be noted that the term “visuo-spatial dyspraxia” has tended to render the differential diagnosis between dyspraxia and CVI almost impossible to achieve. Indeed, visuo-spatial dyspraxia (Mazeau, 2005) includes a certain number of CVIs (reduction of visual field, attention and spatial organization disorders) which are themselves thought to be responsible for gestural clumsiness (Costini et al., 2014a,b). Indeed, in children with CVI, the use of vision may interfere with motor achievement, whereas verbal instructions or performing a task without visual control tends to improve performance (Mazeau, 2005; Chokron and Dutton, 2016), which may explain why children with CVI often choose to reach to the side of where they are looking. At present, it is therefore necessary to review the concept of “visuo-spatial dyspraxia,” since logically, visuo-spatial disorders alone can explain motor awkwardness. Therefore, as recently proposed by some authors (Costini et al., 2014b), the term “visuo-spatial dyspraxia” should no longer be used in children with clear visual and/or spatial cognitive impairments that may alter their gestural production. Instead, in these children, it is essential to assess neurovisual and gestural disorders independently, with and without visual control (i.e., eyes open or closed), and to reserve the term “practical disorders” only for children whose gestural production is similar under both conditions.

#### Cortical or Cerebral Visual Impairment, Social Interactions and Emotional Reactions

In the healthy individual, social interactions are based not only upon the exchange of verbal information but also non-verbal information, especially cues mainly expressed through eye contact, gestures and facial expressions. Even in the context of typical development, it is more difficult for a child than for an adult to analyze and give meaning to facial expressions that convey emotion, but this is even more difficult for those with a CVI. From the first months of life, the visual system thus allows the development of tools that are indispensable for interactions with others (Chokron and Streri, 2012). These include the implementation of purely visual communication and then joint ocular attention, which informs the baby from the age of 9 months (Baron-Cohen et al., 1997) about the location and direction of the individuals in front of him. According to Itier and Batty (2009), joint attention, or shared attention of two individuals on the same object, is one of the prerequisites for the development of Theory of Mind, which allows us to make causal inferences about the behaviors of others, being mature around the age of 4–5 years (Mitchell and Lacohee, 1991).

There is a particular challenge for parents who have to interact with a child whose CVI they are frequently unaware of. Indeed, parents of a child with ophthalmologic visual dysfunctioning are warned of future visual difficulties at an early stage and can adapt their behavior accordingly, by using auditory or haptic modalities instead of visual ones. On the contrary, in the child with a CVI, lack of knowledge of the disorder by the medical profession, the family and the child herself, does not allow the stakeholders to interpret the child’s particular behavior in terms of a potential visual cause, and therefore take appropriate action to cater for the causative visual disabilities (McConnell et al., 2021).

Interacting with a child who does not look at you, does not follow you with her eyes, does not recognize you, and does not smile in response to your smile, without being able to relate this set of behaviors to disorder of visual function, is extremely difficult, and likely to alter early relationships. Unlike the visually impaired child with ocular disorders, whose healthy occipital cortex will progressively reorganize itself to process other sensory information to compensate for the visual disorder (touch to see, air friction analysis, echolocation etc.) (Martin et al., 2016; Norman and Thaler, 2019), the child with CVI does not have healthy unused cortical areas that can directly compensate for the visual function disorder. Adaptation to the CVI cannot therefore take place spontaneously, but is dependent upon targeted customized adapted education and re-education, which can only be put in place once the diagnosis and characteristics of the underlying visual disorder have been established.

Numerous studies have shown that blindness or severe congenital visual dysfunctioning are frequently accompanied by autistic features (with a much higher occurrence than that observed in the general population), raising the question of the link between visual dysfunction and autism (Jambaqué et al., 1998; Sonksen and Dale, 2002). In a very similar way, Garcia-Filion and Borchert (2013) have recently found a considerably higher prevalence of autism spectrum conditions in a population of visually impaired subjects, being up to 25%, compared to the estimated occurrence of 0.6% in the general population. A recent study by Jure et al. (2016) confirms this hypothesis. All these studies strongly suggest that it is not the etiology of blindness that seems to be the cause, but rather the absence of visual perception from birth or very early in life.

Cortical or cerebral visual impairment can interfere with any or all aspects of visual processing, from detection to attention, orientation, exploration, search, spatial localization or recognition of objects, scenes, places or faces (Kelly et al., 2021). As a result, disorders of cerebral visual cognition such as those impairing face recognition, perception of facial expressions, gestures, movement, and the environment in general also hinder development of social and emotional interaction by impairing many of the processes necessary for communication, including acquisition of related language skills (Pawletko et al., 2014).

Although rarely mentioned in the literature, autistic-like conditions may exist in children with CVI and *vice versa* (Freeman, 2010; Tanet et al., 2010; Fazzi et al., 2019). These manifestations can lead to the official diagnosis of PDD despite the known presence of a brain lesion and neurovisual symptomatology. The question of differential diagnosis between sequelae of the spectrum of CVI to cortical blindness and PDD is thus increasingly being raised (Jambaqué et al., 1998; Freeman, 2010; Tanet et al., 2010; Pawletko et al., 2014; Chokron and Dutton, 2016; Fazzi et al., 2019) and it is now necessary to inform practitioners how to elicit the differential diagnosis between these conditions.

Some children with CVI may underestimate or overestimate certain facial expressions, especially negative ones, such as fear, anger or disgust, or confuse them with each other. Some children with facial recognition problems (e.g., prosopagnosia) may sometimes misrecognize, and so behave with strangers as if they know them, or conversely, fail to react appropriately to people they know, such as friends, and even siblings or relatives (Fazzi et al., 2009). These face recognition disorders can lead to serious problems in social interaction, especially if they are misunderstood by others, who interpret the lack of reaction as disinterest and not as a visual disorder, resulting in a genuine interaction difficulty. For some children with CVI, these difficulties in recognition and analysis can be so severe and disabling that they can lead them to isolate, reinforcing the image of withdrawal seen in autistic syndromes. According to recent studies (Freeman, 2010; Pawletko et al., 2014), CVIs have such a significant impact on social skills that it can lead many affected children to be misdiagnosed as having PDD, Asperger syndrome or autism (conditions now labeled under the autism spectrum term) (Jambaqué et al., 1998; Fazzi et al., 2019).

It therefore seems essential to be able to search early and systematically for CVIs in at risk children, in order to be able to treat them as quickly as possible, and avoid the occurrence of interaction and/or cognitive and/or behavioral disorders (Lueck and Dutton, 2015b; McConnell et al., 2021). The ability to make the best possible differential diagnosis between CVI and autism would also help to identify the most appropriate targeted intervention for each child, to bring about salient school adaptations, while providing useful parental guidance to optimally stimulate and teach these children as effectively as possible (Fazzi et al., 2019).

## Implications for Early Detection of Cortical or Cerebral Visual Impairment in Children

The recognition of CVI in children is vital to offer parents, educators and stakeholders, management advice aimed at optimizing motor, cognitive and social development as well as school learning. The need to recognize visual function disorders, whether they are ophthalmological or neurological in origin, is now well established (Fazzi et al., 1999) and must result in the implementation of early interventions to improve the future for these children (Chokron and Dutton, 2016; Rossi et al., 2017; Chang and Borchert, 2020).

Unfortunately, in children with a CVI, a large number of behavioral manifestations can be neglected or misinterpreted if visual disorders of central origin are not considered nor taken into account (Lueck and Dutton, 2015a). At present, failure at school leading to relational difficulties is likely to be systematically interpreted in terms of specific cognitive or behavioral disorders, without consideration of CVI, particularly in the children whose visual acuities are normal (Lowery et al., 2006; Pawletko et al., 2014). At the start of schooling, is often the focus upon the child’s activities, rather than the adequacy of their supporting visual processing, that can delay diagnosis (Cavezian et al., 2010b). The situation for children with additional associated neurological or ophthalmological disorders is equally problematic (West et al., 2021). In particular, the visual pathology can be “the tree that hides the forest.” In this scenario, the child who is already being followed-up for ophthalmic disorders, may not necessarily be subject to a complementary neurovisual assessment (Ego et al., 2015; Lueck and Dutton, 2015b; Chang and Borchert, 2020; McConnell et al., 2021). The diagnosis of CVI is crucial, as this can impact the child’s whole development and their future, because the diagnosis can be confused with other conditions, thereby delaying or even preventing appropriate care. The diagnostic approach includes in-depth structured history taking, precise visual assessment and regular evaluation of those affected to identify the condition, assess the evolution and best adapt management to cater for specific needs at school (Ysseldyke et al., 2009; Salvia et al., 2013; Chang and Borchert, 2020). The assessment process thus varies according to the goal, to identify and characterize other disorders, implement targeted interventions, and make decisions concerning the provision of optimal appropriate educational or vocational services (McConnell et al., 2021).

## Conclusion

The neuropsychological approach combined with structured history taking for CVI allows us to finely describe visual function disorders as well as to characterize their deleterious effect on cognitive, social and motor development. Optimal management (that we are not covering here) is founded on this finely profiled description, and aims at truly enhancing all the capacities of detection, discrimination, analysis, memory, and visual attention, as well as the processes involved in the mental organization and representation of space (Zihl and Dutton, 2015; Chokron, 2018; Chang and Borchert, 2020; McConnell et al., 2021), complemented by skilled teaching of parents and teachers about the unique visual difficulties of each child, and the salient actions that they need to take. Future research in this field will aim to standardize both assessment and management, tenable collection of comprehensive data on the subject, and dissemination of diagnostic and rehabilitative methodologies for the dynamic assessment and management of CVI to bring about optimal learning and development. While awaiting the dissemination of these tools, clinicians must finely assess the visual skills of children, taking care to distinguish between primary disorders on the one hand, and their consequences on the overall cognitive sphere on the other, thus avoiding diagnostic confusion, particularly with autism and intellectual disability. This is crucial as it allows distinction between PDD/ASD and/or intellectual disability as a consequence of a primary CVI. A better understanding or such etiological mechanisms is central to propose appropriate solution as early as possible and to spread knowledge on CVI to all professionals caring for children (McConnell et al., 2021).

Twenty years ago, CVI in children was rarely considered or mentioned. Recently, this condition in its many forms has been extensively researched. It is to be hoped that the coming years will see optimal diagnosis and management, especially for children born in a high-risk context (prematurity, neo-natal hypoxia, and neo-natal stroke and non-accidental head injury) for whom targeted screening for CVI is likely to prove effective and worthwhile.

## Author Contributions

SC: design of the manuscript, writing, and editing. KK: editing final draft. GD: writing and editing. All authors contributed to the article and approved the submitted version.

## Conflict of Interest

The authors declare that the research was conducted in the absence of any commercial or financial relationships that could be construed as a potential conflict of interest.

## Publisher’s Note

All claims expressed in this article are solely those of the authors and do not necessarily represent those of their affiliated organizations, or those of the publisher, the editors and the reviewers. Any product that may be evaluated in this article, or claim that may be made by its manufacturer, is not guaranteed or endorsed by the publisher.
